# Supplementary material for: Decoding an Amino Acid Sequence to Extract Information on Protein Folding
Source: Molecules. 2022 May 7;27(9):3020. doi: 10.3390/molecules27093020 (PMC9106047; doi:10.3390/molecules27093020)
Supplement: Supplementary file 1 [file molecules-27-03020-s001.zip › molecules-1681254-supplementary.pdf]

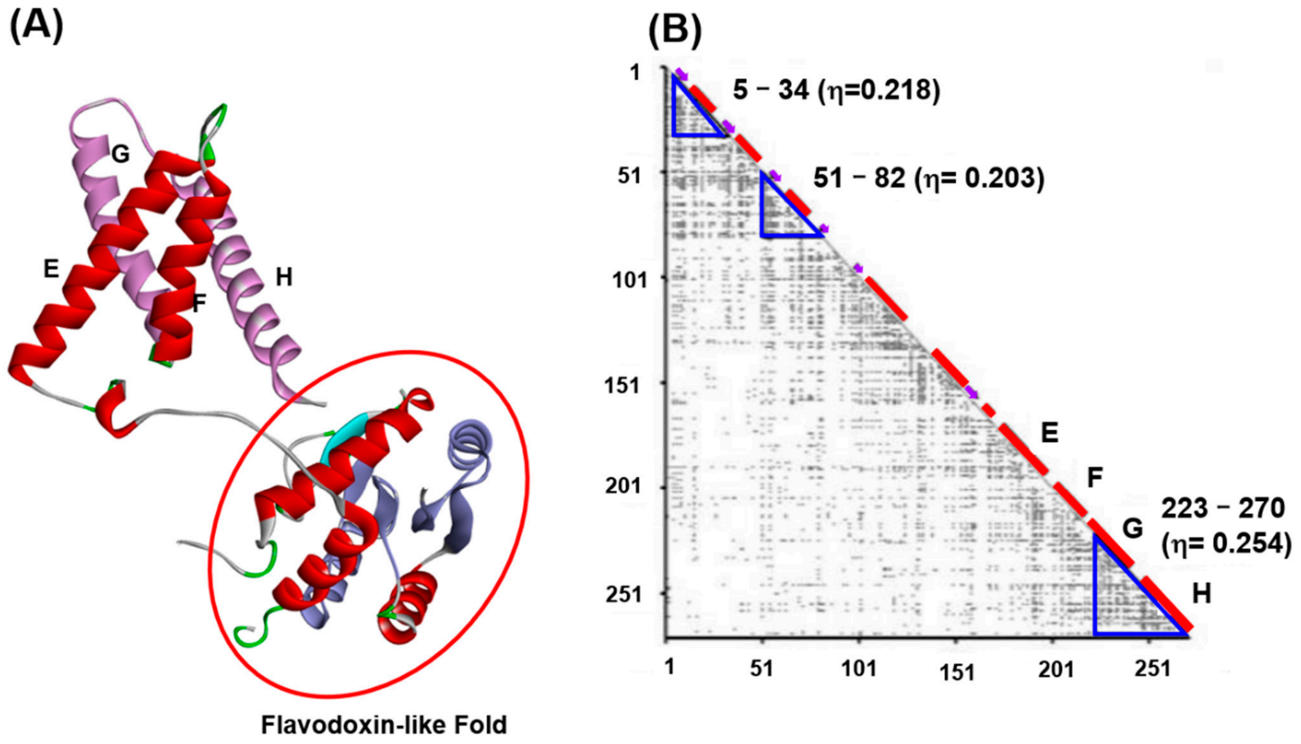

Figure S1. (A) A 3D structure and (B) ADM for the circadian clock protein KaiA (PDB code: 1R8J). The G and H helix part is purple in color. The flavodoxin domain is enclosed by a red circle. A Rossmann fold is blue in color in (A). The ADM predicted regions are enclosed by blue triangles in (B). The segment of a predicted region is labeled near the diagonal of the map. The location of an A-helix is denoted by a red bar on the diagonal. (A β-strand is denoted by a purple arrow in (B)).

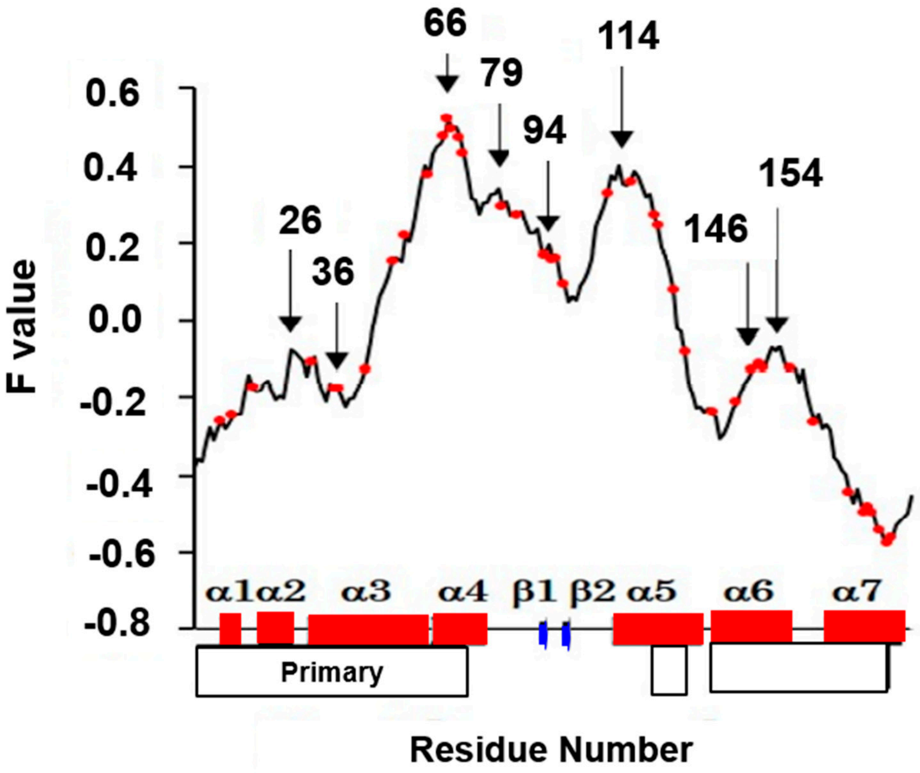

Figure S2 The F-value plot for 153L. A red bar and a blue arrow along the abscissa of a plot indicate the α-helix and β-strand, respectively. An open bar in the bottom of a figure represents a predicted region by ADM. "Primary" means the region with the highest η-value. A red dot indicates a CHR.

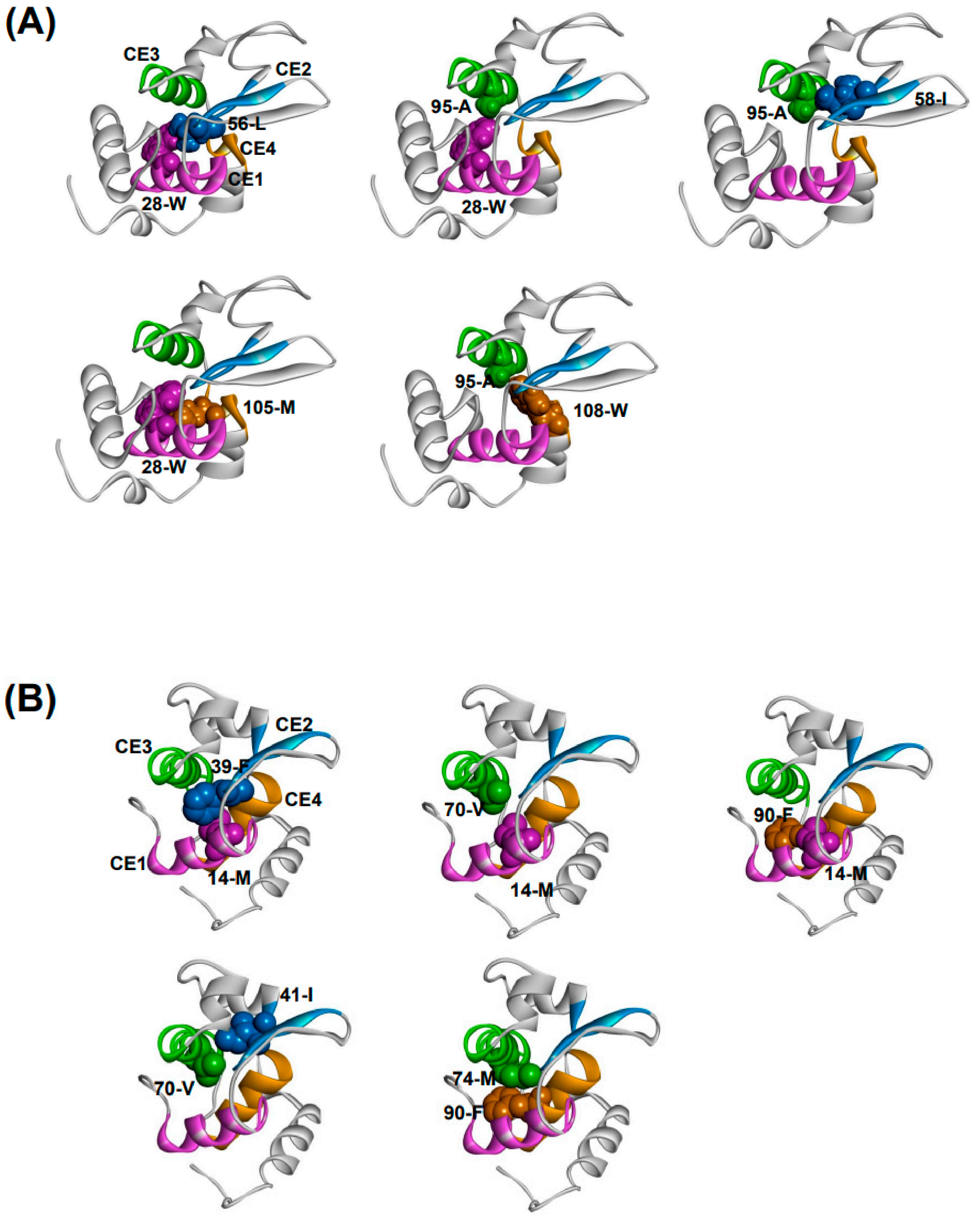

(A)
CE3
CE2
56-L
CE4
CE1
28-W
95-A
28-W
95-A
58-I
105-M
28-W
95-A
108-W
(B)
CE2
CE3
39-F
CE4
CE1
14-M
70-V
14-M
90-F
14-M
41-I
70-V
74-M
90-F

(C)

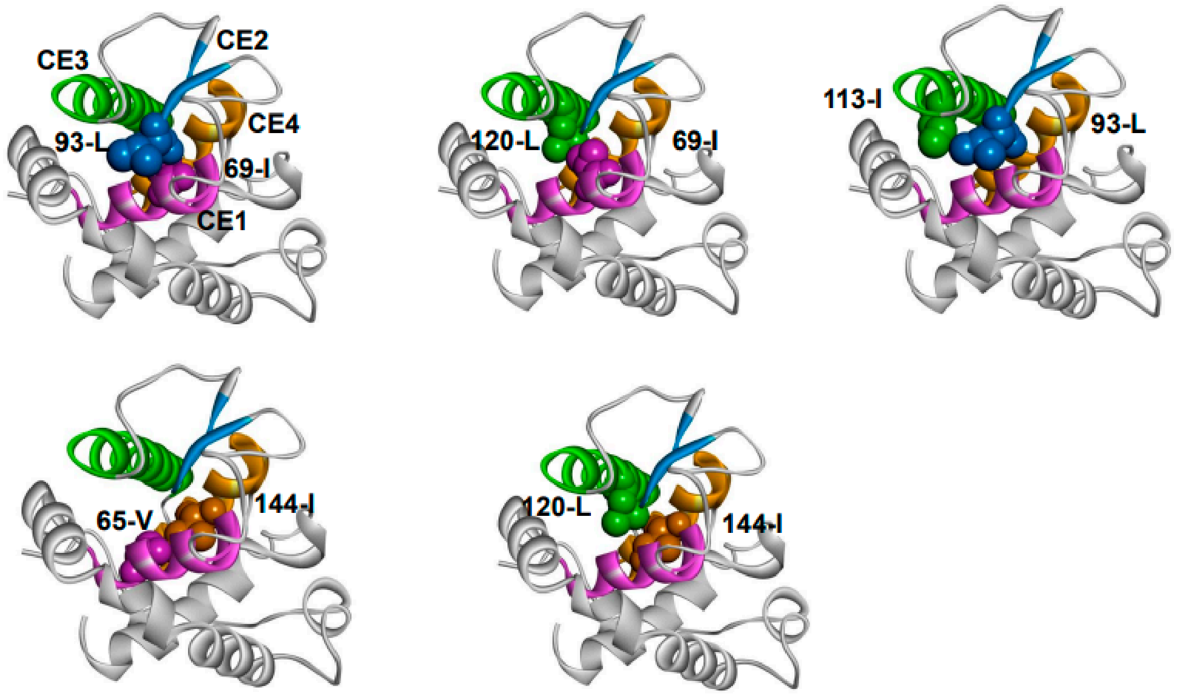

(D)

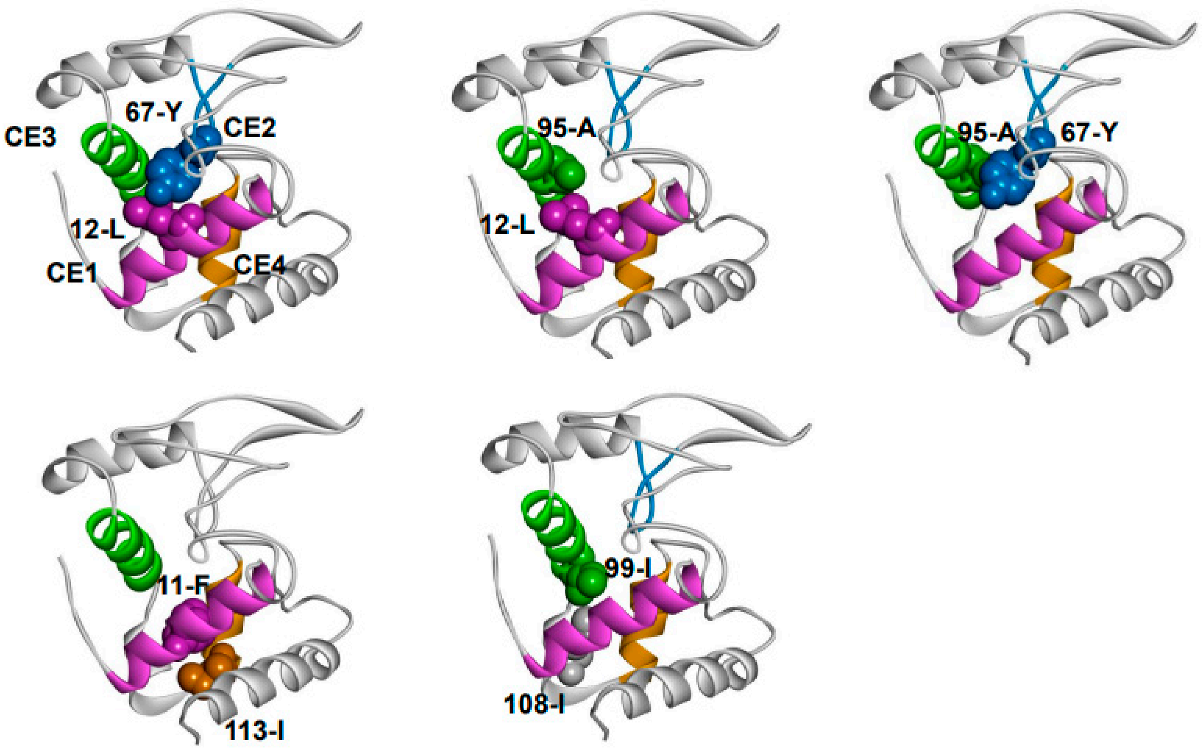

Figure S3: The hydrophobic contacts formed by CHRs in two common secondary structures observed in 2VB1 (A), 2DQA (B), 153L (C) and 1AM7 (D). A secondary structure with the same color denotes a structurally common element (labeled by CE1 (magenta), CE2(blue), CE3(green) and CE4(orange)) in all lysozymes.
